# Clinical Phenotypes, Serological Biomarkers, and Synovial Features Defining Seropositive and Seronegative Rheumatoid Arthritis: A Literature Review

**DOI:** 10.3390/cells13090743

**Published:** 2024-04-24

**Authors:** James Perera, Chiara Aurora Delrosso, Alessandra Nerviani, Costantino Pitzalis

**Affiliations:** 1 Centre for Experimental Medicine and Rheumatology, William Harvey Research Institute, NIHR Barts Biomedical Research Centre, Queen Mary University of London, London EC1M 6BQ, UK; 2 Department of Translational Medicine, University of Piemonte Orientale and Maggiore della Carità Hospital, 28100 Novara, Italy; 3 Department of Biomedical Sciences, Humanitas University & IRCCS Humanitas Research Hospital, 20089 Milan, Italy

**Keywords:** rheumatoid arthritis, seronegative arthritis, synovial tissue

## Abstract

Rheumatoid arthritis (RA) is a chronic autoimmune disorder which can lead to long-term joint damage and significantly reduced quality of life if not promptly diagnosed and adequately treated. Despite significant advances in treatment, about 40% of patients with RA do not respond to individual pharmacological agents and up to 20% do not respond to any of the available medications. To address this large unmet clinical need, several recent studies have focussed on an in-depth histological and molecular characterisation of the synovial tissue to drive the application of precision medicine to RA. Currently, RA patients are clinically divided into “seropositive” or “seronegative” RA, depending on the presence of routinely checked antibodies. Recent work has suggested that over the last two decades, long-term outcomes have improved significantly in seropositive RA but not in seronegative RA. Here, we present up-to-date differences in epidemiology, clinical features, and serological biomarkers in seronegative versus seropositive RA and discuss how histological and molecular synovial signatures, revealed by recent large synovial biopsy-based clinical trials, may be exploited to refine the classification of RA patients, especially in the seronegative group.

## 1. Introduction

Rheumatoid arthritis (RA) is a chronic autoimmune disease characterised by inflammation of the diarthrodial joints. It classically presents as a symmetrical polyarthritis with raised inflammatory markers. Due to systemic inflammation, it can also be associated with other organ complications such as lung fibrosis, scleritis, and lymphoproliferative disease and can contribute to atherosclerosis, leading to strokes and myocardial infarctions [1].

The burden of this condition can be severe, with one-third of patients having to give up their occupation due to the disease within two years of onset [2]. Furthermore, the economic impact of the disease is substantial; in the UK alone, the economic cost of RA (including disability and sick leave) had been estimated at GBP 3.8–4.8 billion per year in 2009 [3,4], the cost of treatment for RA and osteoarthritis (OA) reached GBP 10.2 billion in 2017 [5] and the combined annual costs of sick leave and worklessness due to RA and OA were estimated to be GBP 100 billion in 2019 [6].

The development of “advanced” therapeutics, including biologic drugs and targeted synthetic medications, has offered clinicians further options for treating the disease and has led to significant improvement in patient care. However, despite the advances, only 20% of patients achieve disease remission and up to 40% of patients do not adequately respond to treatment, showing less than 20% improvement in the American College of Rheumatology scores (ACR20) [7]. The identification of patients who remain symptomatic despite conventional treatment has led the European League Against Rheumatism (EULAR) to establish criteria that define “difficult-to-treat RA” [8]. The criteria include problematic signs or symptoms reported by the patient or attending rheumatologist, specific signs that suggest the disease is active, and failure to respond to at least two biological disease-modifying antirheumatic drugs (after failing to respond to conventional disease-modifying antirheumatic drugs) [8].

One of the barriers to treating patients optimally is being able to predict which drug is going to benefit them most. Most guidelines, including UK National Institute for Health and Care Excellence guidelines [9], provide a step-up “trial and error” approach, which leads to several attempts of advanced treatments being tried before an effective drug tailored to the individual patient’s disease is found. This delay may lead to prolonged poorly controlled disease activity with consequent accrual of structural damage to the joints and long-term disabilities. A targeted approach based on personalised patient characteristics is needed to ensure we give the “right drug to the right patient at the right time” [10].

Another challenge encountered by clinicians is the clinical heterogeneity of RA. Patients can present with a variety of clinical manifestations. The identification of autoantibodies such as rheumatoid factor (RF) and anti-cyclic citrullinated peptide (anti-CCP) has helped to diagnose patients presenting with symptoms of inflammatory arthritis as RA. Testing for these autoantibodies has been incorporated into the ACR/EULAR 2010 criteria for diagnosing RA [11]. Out of six points in total to fulfil the criteria for a diagnosis of RA, patients can score three points if they have high titres of either RF or anti-CCP. RA patients who have these autoantibodies have been termed to have “seropositive” RA, while those who have clinical manifestations of RA but lack these antibodies are defined as having “seronegative RA”. The previous ACR 1987 criteria included only RF, as anti-CCP had not been developed yet [12].

While the ACR/EULAR 2010 criteria certainly helped to improve the ability to diagnose patients with RA at an earlier stage to ensure earlier intervention, i.e., before the establishment of radiological damage, when applied to seronegative cohorts of patients, their sensitivity decreases, with Kaneko et al. showing a 95.9% sensitivity in seropositive patients versus a 15.8% sensitivity in seronegative patients [13]. Furthermore, it is commonly thought that seronegative RA patients are less likely to progress and are easier to manage [14], and so clinicians may be less likely to escalate treatment for these patients.

In the drive towards precision medicine, understanding the differences between seronegative and seropositive RA and further characterising clinical phenotypes among the heterogeneous seronegative group will help to stratify patients into distinct clinical endotypes and allow the application of targeted treatment. This would also help to address the “difficult-to-treat RA” patients. This review describes up-to-date differences in epidemiology, clinical features, and serological biomarkers in seronegative versus seropositive RA and discusses how histological and molecular synovial signatures, revealed by recent large synovial biopsy-based clinical trials, may be exploited to refine the classification of RA patients, especially the seronegative group.

For this review, we will define “seronegative RA” as those RA patients who are negative for RF and anti-CCP. RA patients positive for anti-citrullinated protein antibodies (ACPAs) but not RF will be defined as “ACPA-positive RA”, while those positive for RF but not ACPAs will be defined as “RF-positive RA”. “Seropositive RA” will refer to RA that is positive for both ACPAs and RF.

## 2. Epidemiology, Genetics, and Clinical Phenotypes: Similarities and Differences between Seropositive and Seronegative RA

### 2.1. Epidemiology of Rheumatoid Arthritis: What Is Changing?

The prevalence of RA varies between 0.5 and 1% worldwide with a slightly higher prevalence in North American indigenous populations [1]. The incidence usually peaks between the ages of 30 and 50, and the disease is more common in biological females with a ratio of 2–3:1 [1].

There are varying estimates of the prevalence of seronegative RA in the literature, with a recent meta-analysis of biologic registries reporting 20–30% of patients with RA being seronegative [15]. However, given these estimates are derived from drug registries, some authors argue that the true prevalence of seronegative RA remains unclear [16]. Clinicians may be less likely to escalate patients to advanced treatment due to the perception that seronegative arthritis is a less aggressive disease, leading to registries being weighted towards seropositive patients, and consequently any data about the prevalence being skewed [16].

Fluctuations in the incidence of seropositive and seronegative RA have also been noted. Myasoedova et al. analysed medical datasets from the population of Olmstead County in the USA and compared the trends in RA incidence between different decades [17]. Between 2005 and 2014, the incidence of RF-negative RA had increased compared to previous decades (which used the 1987 ACR criteria [12], as opposed to the 2010 ACR/EULAR criteria [10]), with a corresponding decrease in RF-positive RA. Conversely, a study by Muilu et al. [18], including a large Finnish population, described a decrease in the incidence of seronegative RA between 2010 and 2014. However, since this population was derived from a registry of antirheumatic drugs, this cohort may not be fully representative and may be skewed towards a higher number of seropositive patients for the same reasons mentioned above.

The potential rise in seronegative RA noted in Mysoeodova et al.’s study may also be due to an increasing ageing population in addition to using improved classification criteria; Matthijssen et al., for instance, suggested that the rise in the incidence of seronegative RA, detected in a large cohort of RA patients in the Netherlands (the “Leiden Early Arthritic Clinic cohort”), was in part due to an ageing population [19]. This observation is also in keeping with differences in the age of onset found by Carbonell-Bobadilla et al., who reported a higher age at diagnosis in seronegative (54 ± 11 years) vs. seropositive patients (43 ± 14 years) [20]. Furthermore, since cigarette smoking is a well-known enhancer of increased peptidyl arginine deaminase 2, which in turn leads to protein citrullination, an overall reduction in the smoking habits in the population may be an additional explanation of the lower prevalence of ACPA-positive RA [21].

Given that biological females with RA have a worse prognosis [22] and the presence of autoantibodies is also associated with a worse disease outcome, associations between seropositive status and biological sex have been studied. Hadwen et al. [23] performed a meta-analysis of 84 studies with a combined population of 141,381 patients. Unexpectedly, it was found that biological females were less likely than biological males to be seropositive for either RF or ACPAs (adjusted for age, smoking status, body mass index (BMI), and clinical severity scores). Further work is needed to understand whether this is due to unestablished inflammatory pathways in seronegative RA that are chromosomally linked or whether nociceptive pathways, which may differ between men and women [24], lead to a higher proportion of biological females having tender joints and a subsequent diagnosis of RA.

### 2.2. Genetic and Environmental Factors Differences

The greatest risk factor for developing RA is a family history, with a first-degree relative with RA increasing the risk by a factor of 2 to 5 [1]. Therefore, although the pathogenesis of RA is not fully understood, a genetic component is likely involved. The human leucocyte antigen (HLA) locus has been long known to be associated with RA [25]. In particular, mutations in the HLA-DRB1 gene have led to the shared epitope hypothesis, whereby a certain sequence of amino acids found in several alleles is thought to abnormally affect the presentation of proteins to T cells, driving autoimmune degradation of self-antigens in the joints [25,26].

Genetic differences between seropositive and seronegative RA can be divided into HLA and non-HLA differences. Alleles for HLA-DRB1*04 and HLA-DRB1*10 have been reported as risk factors for the development of seropositive RA [25]. Evidence for HLA association with seronegative RA is less strong; however, the HLA-B08/DRB103 haplotype has been reported to be more associated with seronegative RA [26,27]. Non-HLA genetic differences have been derived from genome-wide association studies (GWAS) Seronegative RA is associated with single-nucleotide polymorphisms (SNPs) in the *CLYBL* gene encoding for a “citrate lyase subunit beta-like protein” [28] and the *ANKRD55* gene, the biological function of which is not clear [29].

Interestingly, variants of genes for the Janus kinase/signal transducer and activator of transcription (JAK/STAT) pathway, the target of the advanced therapeutics JAK inhibitors, have been found to be risk factors for developing seropositive but not seronegative RA [30]. However, as of yet, there is no evidence that seropositivity affects the treatment outcomes of JAK inhibitors [31,32].

A large cohort trial (*n* = 88,639) has reported that the heritability of RA in seropositive RA is 50%, compared to 20% in seronegative RA, suggesting that genetic influences in the pathogenesis of RA may be more relevant in the seropositive group [33]. A review by Padyukov on the genetics of RA concluded that polymorphisms involved in the pathogenesis of RA are better reported in seropositive RA, and that larger studies are needed to perform GWAS of seronegative RA to further clarify the relevance of genetic factors in this population [26].

Several environmental factors contribute to RA pathogenesis. In addition to smoking, previously mentioned in Section 2.1, it is becoming more apparent that the diversity of the genes of intestinal bacteria, known as the gut microbiome, is involved in the evolution of molecular mechanisms that underpin adaptive immunity [34]. Imbalanced homeostasis of the gut microbiome may lead to the production of pro-inflammatory cytokines such as Interleukin (IL)-17a and Tumour Necrosis Factor alpha (TNFα) [35]. Several gut bacteria such as Prevotella species and Collinsella species have been proposed to be implicated in the pathogenesis of RA [36,37].

The association between the microbiome and the development of autoantibodies in RA is of increasing interest. A Chinese study found increased proportions of Clastridiales, Blautia, and Akkermansia species in ACPA-positive RA patients compared to ACPA-negative RA patients [38]. A subsequent small cohort study focussing on bacterial species in RA found that butyrate-producing species were lower in ACPA-negative RA and that butyrate-consuming species were significantly higher in ACPA-positive RA patients [39]. The significance of these results needs further investigation as these were small studies in local populations. Further work is needed to establish if these findings extend to other populations.

### 2.3. Immunopathogenesis of Synovial Inflammation in Seropositive Versus Seronegative RA

The immunopathogenesis of RA is not fully understood, but significant progress has been made. For example, analyses of gene expression and single-nucleotide polymorphisms (SNPs) have found multiple differences between seropositive and seronegative RA, and these can broadly be divided according to cell types [40].

Depending on their in vivo environment, macrophages can differentiate into various inflammatory statuses, some of them showing an M1-like phenotype, which is pro-inflammatory and characterised by the production of cytokines such as TNFα and IL-6 [41]. Using single-cell RNA sequencing of synovial tissue, Wu et al. [42] analysed the differences in ACPA-positive versus ACPA-negative RA. They found that in ACPA-negative patients, there were greater numbers of M1-like macrophages, characterised by absent gene expression of CD36 and transforming growth factor-beta (TGF1B). Furthermore, they found that both macrophages and dendritic cells in ACPA-negative RA had significantly upregulated gene expressions of chemokine ligand 13 (CCL13), matrix metalloproteinase-3 (MMP3), and chemokine ligand 18 (CCL18). Serum levels of CCL18 and CCL13 may be involved in immune cell infiltration of the synovium and cause proliferation and angiogenesis [43,44,45]. MMP3 is an enzyme that is involved in the destruction of joints in RA and has been shown to be an early marker of erosions [46].

Upregulation of certain chemokines and cytokines can contribute to macrophage activation and neutrophil infiltration in RA. Granulocyte macrophage colony-stimulating factor (GM-CSF), for example, induces the overexpression of TNFα, MMP12, and activin A, which stimulate synovial macrophages to become inflammatory macrophages [47]. CCL21 is increased in the RA synovium [48] and is thought to cause monocytes to infiltrate the joint and differentiate into pro-inflammatory macrophages, which subsequently causes the proliferation of osteoclasts [49]. Interferon-regulatory factor 5 (IRF-5) has been associated with neutrophil infiltration into the synovium, and in a large study focussing on genetic variants of IRF-5, SNPs were found to be more associated with ACPA-negative arthritis [50].

In both seropositive and seronegative RA, there is overexpression of genes associated with T-cell differentiation [51]. SNPs within *STAT4*, which is linked to the differentiation of T-helper (Th) 1 cells, and attenuation of *PTPN22*, which leads to citrullination and subsequent increases in Th17 and Th2 cytokines, have been found in both ACPA-positive and -negative RA [51]. It appears that increased levels of IL-6 instigate the signalling of STAT 3 in CD4 + T cells in early RA, and this has been found to be increased in ACPA-negative RA compared to seropositive RA [30,51].

Moreover, synovial tissue CD4+ T cells appear to be more pro-inflammatory in ACPA-negative patients compared to ACPA-positive patients, with higher levels of TNFα expression being observed [52]. A recent study by Alivernini et al. reported that CD138+ plasma cells in the synovial tissue were higher in ACPA-negative RA than in psoriatic arthritis [53]. The reverse was true for mast cells, with psoriatic arthritis (PsA) being mast cell-rich (CD117+) and ACPA-negative RA being mast cell-poor. Decreased infiltrating lymphocytes were found in ACPA-negative patients compared to ACPA-positive patients when examining histology [54].

Fibroblast-like synoviocytes (FLSs) produce matrix metalloproteinases, which degrade collagen and facilitate cell migration, including FLS migration into the joint [55]. FLSs also indirectly cause recruitment of immune cells to the synovium by upregulating endothelial expression of adhesion molecules [55]. FLSs secrete chemokines such as CXCL12 and CCL2, as well as cytokines like IL-6 and pro-angiogenic factors, which ultimately lead to the synovial infiltration of immune cells and therefore inflammation [55,56]. As more and more studies focus on in-depth phenotyping of single cells in synovial tissue, it will be of interest to see how FLSs differ between seropositive and seronegative RA.

### 2.4. Clinical Features and Long-Term Outcomes: Is Seronegative RA a Different Disease?

There are conflicting data in the literature regarding clinical differences between seropositive and seronegative RA. Multiple studies have suggested that seropositive RA is a more severe disease than seronegative RA [57,58,59]. Prior to the introduction of biologics as a therapeutic option, an observational study including over 900 patients from a primary care incidence registry (Norfolk arthritis registry) suggested that the presence of ACPAs was associated with more severe disease at baseline and at 5-year follow-up. It was also found that ACPA-positive patients were less likely to benefit from disease-modifying antirheumatic drugs (DMARDs) compared to ACPA-negative patients. Overall, these findings suggested that a more aggressive escalation of treatment was required for ACPA-positive patients to control their disease [60].

However, in 2017, Nordberg et al. [61] found that seronegative patients had significantly higher clinical and ultrasound (US) evidence of inflammation when comparing US scores and clinical parameters of more than 200 DMARD-naïve patients diagnosed with RA. According to the authors, this somewhat unexpected observation may be linked to the fact that seropositive patients were referred earlier in view of their seropositive status, especially after the introduction of the 2010 ACR/EULAR criteria that gave more weight to the antibody status, and, consequently, had less inflammation than the diagnosed seronegative patients, who were instead referred only when affected by a significantly higher level of joint inflammation.

The extra-articular manifestations of RA have also been found to differ between seropositive and seronegative patients; for example, scleritis and rheumatoid nodules are more likely to be present in seropositive patients [62,63]. Similarly, a meta-analysis reported that ILD was more associated with higher titres of anti-CCP antibodies [64]. RA is generally associated with a higher risk of cardiovascular disease, most likely due to chronic inflammation that causes increased arterial stiffness and vascular changes [65]. A study investigating changes in carotid arteries found that seronegative patients have significantly increased ultrasound markers suggestive of atherosclerosis compared to seropositive patients, suggesting they may have more inflammatory activity leading to atherosclerosis [66].

Differences in the response to treatment between seropositive and seronegative RA are also subject to debate. Methotrexate appears to be less effective in seronegative populations [67,68], but when analysing responses to biologic drugs, studies suggest that responses are similar between seronegative and seropositive RA patients [69,70]. A recent study by Jin et al. [71] compared the effectiveness of biological DMARDs and JAK inhibitors between more than 4000 seronegative and more than 7000 seropositive patients. They found that the clinical effectiveness, assessed 12 months after starting treatment, was not significantly different between the two groups.

This observation should be taken in the context of the still relatively high numbers of patients who do not achieve a meaningful response to treatment. As detailed below (Section 4.1), over the last twenty years, our group and others have focussed on identifying synovial histological and molecular biomarkers of response to treatment to “personalise” the therapeutic approach for patients with RA. Although synovial tissue markers able to differentiate seropositive and seronegative patients are yet to be defined, these could potentially be considered in future stratified trials to better understand the mechanisms of response/non-response in each patient group.

Overall, given the development of new drugs over the last two decades, we have certainly witnessed an improvement in the long-term outcomes of RA patients. Matthijssen et al. [72] studied the long-term outcomes of 1285 patients with RA between 1993 and 2016 and specifically focussed on analysing differences between seronegative and seropositive RA. While disease activity improved over time in both groups, functional disability and mortality only significantly improved in seropositive RA patients. This is particularly concerning and suggests further work is needed to improve these outcomes in seronegative RA.

### 2.5. Radiological Differences and Erosive Burden

Multiple studies have analysed the relevance of seropositive status to the appearances of the joints in different modalities of imaging.

#### 2.5.1. X-ray

Bone erosion is a radiological term that refers to when bone resorption outweighs bone formation, leading to breaks in the periarticular cortical bone surface that can be seen on X-rays [73]. While many conditions can cause bone erosions, the commonest cause is synovitis in RA. At present, bone erosion is thought to be irreversible and can occur early in RA, with the majority of untreated patients developing erosions within 2 years [74]. The presence of bone erosions was included in the previous 1987 ACR criteria for diagnosing RA [12]; however, it was removed from the 2010 ACR/EULAR criteria as it was felt that patients who could be treated effectively prior to the development of erosions were being excluded [11].

Several studies in the 2000s showed significant associations of both RF and ACPAs with radiographic progression on X-rays [75,76,77]. Forslind et al. performed a detailed statistical analysis on the relationship between ACPAs, RF, and radiological joint damage and found that ACPAs were the best independent predictor of radiological damage [78].

However, the exact role of ACPAs in determining bone erosion is unclear. One hypothesis is that synovitis is the prime driver of the production of chemokines that attract immune cells capable of developing into osteoclasts to the synovium. Another hypothesis is that plasma cells in the bone marrow produce ACPAs that activate osteoclasts, causing erosions [73,79]. A recent study by Di Matteo et al. investigated the presence of bone erosions in relation to ACPA-positive patients with musculoskeletal symptoms, but with no clinical synovitis [80]. They found that without synovitis, the presence of bone erosions was very low in ACPA-positive patients and that bone erosions did not predict the development of inflammatory arthritis. Notwithstanding the possible role of ACPAs in driving erosions, given that historically, ACPAs were associated with more severe synovitis, the relationship between ACPA positivity and bone erosions may be reflective of the relationship between synovitis and bone erosions. Therefore, a subgroup of seronegative RA patients with severe synovitis may be at greater risk of developing erosions and may require greater vigilance and earlier treatment escalation.

#### 2.5.2. Ultrasound

Ultrasound is more accurate than clinical examination in detecting synovitis and has been shown to be able to help differentiate between seronegative RA and OA [81,82]. Standardised ultrasound scoring systems have been developed to facilitate its use in clinical trials [81], and some studies have compared US features between seropositive and seronegative RA.

Lin et al. compared the ultrasound scores of a Chinese cohort of DMARD-naïve patients with RA (*n* = 139) [83]. When adjusted for the number of joints involved, they found that seronegative patients had significantly milder inflammation. On the other hand, a separate study by Ruta et al. found that seronegative patients had significantly higher amounts of tenosynovitis (*n* = 746) [84]. Interestingly, tenosynovitis is a typical feature of psoriatic arthritis.

Further studies are needed to confirm these findings and establish whether ultrasound can be used to identify phenotypic features rather than just confirming the presence of inflammation.

#### 2.5.3. Magnetic Resonance Imaging (MRI)

Even in patients deemed to be in clinical remission, MRI is able to detect subclinical synovitis and can predict radiographic progression of the disease [85]. The use of MRI has particular relevance to seronegative RA; Den Hollander et al. [86] investigated the predictive value of MRI in RA and found that RA progression was associated with MRI-detected tenosynovitis in seronegative patients with oligoarthritis. They concluded that a negative MRI in the seronegative population could be used to prevent overtreatment.

Advances in machine learning are increasingly becoming more relevant to MRI analysis [87]. A recent study by Folle et al. [88] found that neural networks that have been trained on MRI imaging are able to distinguish between seropositive RA, seronegative RA, and psoriatic RA. This was particularly intriguing, as the addition of clinical data to the network did not improve classification, suggesting that MRI can pick up pathological differences in disease without clinical correlation. Although this was a preliminary study and the results need to be replicated, this exciting new technology may provide us with a better understanding of the pathological mechanisms underpinning these diseases and how they differ.

## 3. The Role of Peripheral Biomarkers in Rheumatoid Arthritis: From Autoantibodies to microRNA

The quest for testable autoantibodies in RA has been ongoing for the last few decades [89]. Despite numerous autoantibodies being discovered and improvements in the sensitivities and specificities of current tests, the need to diagnose and treat seronegative patients earlier propels the search for new testable autoantibodies forward. Here, we review the current literature regarding testable autoantibodies in RA. We summarise the main insights in Table 1.

### 3.1. Rheumatoid Factor and Anti-CCP Antibodies

Rheumatoid factor and anti-CCP antibodies are the conventional antibodies routinely tested by clinicians when they suspect that a patient has RA. The historical development of these tests is important in understanding why they are used in clinical practice, but recent research also proposes novel methods to use them more precisely.

#### 3.1.1. Rheumatoid Factor (RF)

RF is an antibody which targets the crystallisable fragment (Fc) portion of immunoglobulin class G (IgG) antibodies [90]. RF itself can exist as different isotypes (IgM, IgG, and IgA). The main isotype is IgM [90], but the IgA isotype has been associated with sicca syndrome and a more erosive disease phenotype [91]. There is variation in the literature on the sensitivity and specificity of RF in diagnosing RA, with sensitivity ranging between 60 and 70% and specificity varying between 50 and 90% [90,92]. RF can be raised in infectious diseases and other autoimmune diseases such as Sjogren’s, but also in healthy subjects [93]. In healthy individuals, the presence of IgM RF has been found to increase with age [94].

First discovered in the serum of RA patients in 1937 [95], RF has been extensively studied, but the mechanism of its production or its involvement in the pathogenesis of RA is still not fully understood. It is thought that in healthy individuals, RF may be involved in the clearance of immune complexes, binding to antibodies that have bound to invading pathogens [96]. In RA, RF present in the joint may induce macrophages to produce pro-inflammatory cytokines [97].

Using novel molecularly engineered IgG targets to study potential epitopes targeted by RF, Falkenburg et al. emphasised the role of epitope spreading in pathogenicity, noting that RF antibodies that are broad (i.e., targeting multiple epitopes) are more likely to lead to the development of RA [96]. Additionally, they were able to distinguish RF between RA patients and patients with Sjogren’s syndrome [96]. The use of these techniques rather than conventional serum RF tests may improve specificity by identifying RA-specific RFs, as well as sensitivity by identifying low levels of RA-specific RFs that do not meet the cut-off criteria of current tests.

#### 3.1.2. Anti-Cyclic Citrullinated Peptide Antibodies

The initial lack of specificity of RF led to the search for more targeted antibodies in the latter half of the 20th century. Multiple antibodies associated with RA, including anti-filaggrin antibodies, antikeratin antibodies, and antiperinuclear antibodies, were proposed as potential candidates [98]. It was found that the epitopes recognised by these antibodies were citrullinated, a process where arginine is converted to citrulline via peptidyl-arginine deaminases (PADs) [93]. This discovery led to the development of a test in the 1990s that was able to detect a cyclic citrullinated peptide that was derived from human filaggrin. The test was improved, and the specificity of the test in relation to RA improved to 98–99% [89,99]. It is worth noting that the cyclic citrullinated peptide is not necessarily the target for autoantibodies in RA, but a biomarker that infers citrullination is taking place. Filaggrin, fibrinogen, collagen type II, and vimentin have been proposed as possible citrullinated targets for ACPA [93,100,101].

In addition to the genetic associations described earlier (Section 2.2), multiple environmental factors have been hypothesised to contribute to citrullination. Smoking is strongly associated with the development of ACPA-positive RA, and citrullinated proteins found in the bronchoalveolar lavage fluid of smokers (but not non-smokers) have led to the theory that smoking stimulates protein citrullination and subsequent production of ACPAs [102,103]. Bacteria involved in the development of periodontitis, such as porphyromonas gingivalis, are also associated with citrullination [104]. Antibodies to this bacterium correlate with ACPA levels, and it is thought that porphyromonas gingivalis produces PAD enzymes that facilitate citrullination [104].

The anti-cyclic citrullinated peptide has been well described in the literature and has also been included in the 2010 ACR/EULAR criteria for RA [11]. Further work performing structural analyses of ACPAs led Ge and Holmdahl to propose a spectrum of ACPAs [105]. On one end of the spectrum, ACPAs are termed “private”, meaning they recognise citrulline and a proximal amino acid, leading to interactions with specific proteins. Private ACPAs cross-react with specific proteins in the joint and can lead to arthritis. On the other end of the spectrum, ACPAs are termed “promiscuous” as they recognise a broad array of citrullinated peptides. These ACPAs tend to be present in the serum before the development of RA and, to date, there is no evidence that they are pathogenic [105].

While the presence of ACPAs can be observed prior to developing RA, there are few studies focussing on serial ACPA titres and seroconversion [106]. Burr et al. [107] analysed ACPA and RF measurements of 640 patients with inflammatory arthritis at baseline and at 5 years and concluded that repeat testing did not improve prognostication and advised against repeat testing routinely.

### 3.2. Novel Biomarkers in “Seronegative” Arthritis

A lingering question regarding seronegative RA is whether it truly is “seronegative”. We have defined it so far as the absence of RF or ACPA; however, the presence of other biomarkers, including antibodies, that are not conventionally measured in RA patients may call this definition into question. Here, we review novel biomarkers that have been proposed to characterise seronegative RA patients. 

#### 3.2.1. Anti-Carbamylated Protein Antibodies

Anti-carbamylated protein (anti-CarP) antibodies have been reported to occur in ~25–50% of RA patients, independently of positivity for RF or ACPAs [108]. These antibodies target carbamylated proteins and lead to the production of homocitrulline, which is similar in structure to citrulline. Carbamylation can occur when isocyanic acid reacts with amino groups of lysine, which converts it to homocitrulline via the addition of a carbomoyl group. Isocyanic acid is derived from cyanate, thiocyanate, or urea. Patients with renal disease have raised urea, which leads to higher levels of cyanate and, therefore, increased carbamylation. Similarly, smoking also increases cyanate, leading to increased carbamylation [92]. Currently, enzyme-linked immunosorbent assays use carbamylated foetal calf serum as a substrate for anti-CarP antibody tests [109]. Specific targets of anti-CarP include carbamylated fibrinogen [110], specifically human fibrinogen β chain [111]. Further work by Brevet et al. [109] identified that the α and γ chains of fibrinogen may also be targets of anti-CarP. Investigating the molecular make up of anti-CarP, van Delft et al. reported that a range of anti-CarP subclasses could be detected, including all IgG subclasses, as well as IgM and IgA [112].

The sensitivity of this antibody to detect RA has been reported as 45% with a specificity of 90% [71]. Furthermore, it has been found to be present prior to the onset of RA [113], and can be used to predict progression to the disease as well as joint erosions [114,115]. A meta-analysis of 12 studies concluded that when anti-CarP antibodies were present in addition to RF and ACPAs, the specificity for RA increased to 98–100% from 65–100% (RF and ACPAs alone). A proteomic approach by Sidiras et al. [116] involved performing mass spectrometry on synovial fluid and serum. Of 11 novel carbamylated proteins found, 5 were considered potential autoantigens, and by using these in conjunction with ACPAs and RF, 89% of a cohort of early RA could be identified.

These antibodies provide an alternative pathway to citrullination that could underlie the pathogenesis of seronegative RA. Studies are needed to assess whether routine testing for anti-CarP will facilitate earlier diagnosis of RA in those lacking RF and ACPA.

#### 3.2.2. Other Autoantibody Candidates

Autoantibodies that target peptidyl-arginine deaminase (PAD) enzymes have been found to be present in 35% of patients with RA [117]. The PAD enzymes are involved post-transcriptionally in the deamination of arginine to citrulline, and there are five different isoforms of PAD enzymes (PAD1, PAD2, PAD3, PAD4, and PAD6). Anti-PAD4 antibodies have been associated with radiographic progression as well as increased severity of disease [117,118,119]. Similarly, the presence of anti-PAD3 antibodies characterises a subgroup of RA patients with a more severe disease radiographically [120,121]. Consistently, a large study by Lamacchia et al. confirmed that anti-PAD3 antibodies were associated with increased joint damage scores and disease activity in RA patients [122]. Like other autoantibodies, anti-PAD3 antibodies can be detected in the blood before the clinical onset of RA [123]. Conversely, anti-PAD2 antibodies have been associated with a milder form of the disease [124]. It is hypothesised that these autoantibodies increase calcium sensitivity, which, in turn, activates the PAD enzymes, causing a feed-forward mechanism where the activated PAD enzymes induce citrullination, generating autoantigens and leading to further autoantibody production [121].

Moore et al. hypothesised that anti-mitochondrial antibodies (AMAs) may be involved in the pathogenesis of RA [125] as extracellular mitochondria have been found in the plasma of RA patients [126]. Anti-mitochondrial antibodies were measured in three cohorts of RA patients and healthy controls, with disease progression being monitored over 8 years. The authors found that AMAs were significantly raised in RA patients in comparison to controls and were associated with interstitial lung disease and erosive disease. Furthermore, it was suggested that AMA levels could be used to predict erosive disease, independently of the “conventional” seropositive status (RF and ACPA). Additionally, antibodies that target the outer membrane protein of mitochondria (anti-mitofusin-1 antibodies) were found to be able to identify seronegative patients who were developing erosive disease [125].

Tissue damage and oxidative stress cause the production of advanced glycation end-products (AGEs) [127] as well as malondialdehyde–acetaldehyde adduct-modified proteins [128]. Autoantibodies can develop against these targets [128,129]. To define additional antibodies implicated in the pathogenesis and progression of RA, van den Beukel et al. analysed 648 patients with RA and 538 patients without RA and detected these antibodies in ~45% of patients with RA and 30% of controls. Of note, these antibodies were associated with radiological progression in the “seronegative” RA group [130].

Recognising the need for novel biomarkers in seronegative RA, Li et al. attempted to find suitable candidates by screening high-density protein microarrays [131]. They subsequently validated candidate autoantigens using ELISA and Western Blot analyses. Of the nine candidates identified, anti-PTX3 and anti-DUSP11 had the highest sensitivity.

The recent development of immune checkpoint inhibitors (ICIs) has been a great success in treating cancer patients [132], but one of the side effects observed has been the onset of autoimmune diseases [133]. Inflammatory arthritis with features resembling RA is among the autoimmune conditions that develop following exposure to these treatments, with most of these patients being seronegative [134]. Anti-RA33 antibodies target a nuclear protein that is involved in the splicing of mRNA. It has been previously found in seronegative RA patients, and Cappelli et al. hypothesised it may be involved in the development of RA following ICI therapy [135]. They found that ~11% of patients affected by ICI-induced arthritis had anti-RA33 antibodies, compared to 0% of patients who received ICIs but did not develop inflammatory arthritis. It was therefore suggested that anti-RA33 could be used as a biomarker to establish the risk of developing inflammatory arthritis after ICI therapy. A meta-analysis of studies reports the use of anti-RA33 as a biomarker for RA to be specific (90.1%) but not sensitive (31.8%). Therefore, the use of anti-RA33 may not be confined solely to post-ICI therapy.

#### 3.2.3. Long Pentraxin 3

Long pentraxin 3 (PTX3) is a protein that has been found to increase in inflammatory states. Unlike C-reactive protein, which is produced by cells in the liver, PTX3 is produced in a variety of cells including myeloid and endothelial cells as well as fibroblasts in response to TNF-alpha and IL-1 [136,137].

PTX3 has been found to be higher in RA patients compared to healthy controls [138], and synovial fibroblasts in RA patients have been shown to produce PTX3 [139]. The use of PTX3 as a marker of disease activity depended on what cohorts were being studied, with some studies showing an association with radiographic disease progression [140] and C-reactive protein (CRP) [141] and other studies showing no significant association with clinical parameters [142,143].

It is plausible that these differences result from differences in patient cohorts, such as stage of disease and, particularly, drug exposure. To minimise the impact of these factors, we studied serum and synovial samples from RA patients at baseline and 6 months after DMARD exposure. RNA sequencing of synovial tissues showed that transcriptomic levels of PTX3 strongly correlated with disease activity scores (DAS28) and CRP [136]; we also found that synovial transcriptomic expression was significantly higher in seropositive patients compared to seronegative patients. This correlated with findings by Weitoft et al. who found that seropositive RA patients had significantly greater levels of PTX3 in synovial fluid when compared to seronegative RA patients [144].

#### 3.2.4. Peripheral Blood MicroRNA

Small single-stranded RNAs that are noncoding are known as “microRNA” (miRNA) and have been found to be important in the regulation of genes post-transcription [145]. They bind to the 3’ untranslated region of messenger RNA and cause transcript destabilisation or mRNA degradation, leading to the silencing of a gene and subsequent protein suppression [145]. In certain cases, miRNA can stimulate gene expression [146]. The expression of 60% of encoding genes is regulated by miRNA, and, therefore, they represent an important target in understanding epigenetic variation [147].

Multiple studies have reported miRNAs involved in pro-inflammatory signalling pathways in RA patients leading to increased production of inflammatory cytokines [148,149,150,151]. A recent study by He et al. compared different miRNAs between seropositive RA, seronegative RA, and healthy controls [152]. Interestingly, differences between seropositive and seronegative patients were found, with hsa-miR-362-5p more upregulated in the seronegative RA group, and hsa-miR-187-3P and hsa-miR-6855-5p being upregulated in the seropositive group. This was a small study, so further work is needed to corroborate these findings, but if true, the authors argue that hsa-miR-362-5p could be used as a potential biomarker for seronegative RA.

All the above studies, which we have summarised in Table 1, need replication and further validation in different cohorts. A proportion of seronegative RA patients may be explained by these antibodies, but, as of yet, wide-scale testing is not available. Therefore, other methods to stratify RA patients are needed.

**Table 1:** Novel biomarkers in rheumatoid arthritis.

Novel Biomarkers in Rheumatoid Arthritis	Available Statistics	Target	Features and Phase of Disease
Anti-carbamylated protein antibodies (anti-CarP) [71,108,110,113,114,115,116]	- Sensitivity: 45% - Specificity: 90% - When present in addition to RF or ACPAs, specificity increases to 98–100%	Carbamylated proteins	- Can be found prior to onset of RA - Can be used to predict progression to disease
Anti-Peptidyl-Arginine Deaminase Antibodies (anti-PAD) [117,118,119,120,121,122,123,124]	- Found in 35% of patients with RA	Peptidyl-arginine deaminase enzymes (5 different isoforms)	- Anti-PAD2 antibodies associated with milder form of disease - Anti-PAD3 antibodies and Anti-PAD4 antibodies associated with a more severe disease radiographically - Anti-PAD3 antibodies can be detected in blood prior to clinical onset of RA
Anti-mitochondrial antibodies (AMAs) [125,126]	n/a	Extracellular mitochondria	- AMA levels have been used to predict erosive disease independently of RF/ACPA status. - Anti-mitofusin-1 antibodies are able to identify seronegative patients who are developing erosive disease.
Anti-RA33 [135]	- Sensitivity 31.8% - Specificity 90.1%	RA33—a nuclear protein involved in splicing of mRNA	- May be helpful post-immune checkpoint inhibitor therapy to establish risk of developing inflammatory arthritis
Antibodies against advanced glycation end-products and malondialdehyde–acetaldehyde adducts (Anti-AGE and MAAs) [128,129,130]	n/a	- Glycation end-products and malonidaldehyde–acetaldehyde adducts	- Associated with radiological progression in seronegative RA
Peripheral Blood MicroRNA [152]	n/a	n/a	- Hsa-miR-362-5p more upregulated in the seronegative RA group - Hsa-miR-187-3P and hsa-miR-6855-5p upregulated in seronegative RA group
Long pentraxin 3 [136,144]	n/a	n/a	- Significantly higher levels of long pentraxin 3 in seropositive RA compared to seronegative RA

## 4. Synovial Tissue Histopathology and Relationship with Autoantibody Status

### 4.1. Synovial Tissue in RA

#### 4.1.1. Histological Pathotypes and Immuno-Serological Status

The hallmark of RA is the chronic inflammation of the synovial tissue. Therefore, the in-depth analysis of the “joint” tissue could help us to further understand the pathogenesis of the disease, refine the classification, and predict the prognosis and response to treatment. Historically, the gold standard for acquiring synovial tissue is via arthroscopy [153]. However, over the last two decades, the pioneering of ultrasound-guided synovial biopsies (with a guillotine-type semiautomatic needle or a portal and forceps system) has allowed sufficient samples to be yielded via a minimally invasive approach [154]. The development and availability of such sampling techniques have been critically important to fuel the research into synovial tissue biomarkers.

Our group and others have focussed on the analysis of histological features of the rheumatoid synovium aiming to identify predictors of disease progression. Since 2008, the “Pathobiology of Early Arthritis Cohort” (PEAC) (https://peac-mrc.mds.qmul.ac.uk accessed on 15 January 2024) has been recruiting patients with early (symptoms < 12 months) inflammatory arthritis naïve to steroids and DMARDs [155]. Patients undergo US-guided needle synovial biopsies of the most inflamed joint prior to starting treatment and 6 months afterwards.

The histological assessment of the synovial tissue of large cohorts of patients allowed the identification of at least three “histological patterns” defined according to the presence and distribution of CD68+ macrophages, CD20+ B cells, CD3+ T cells, and CD138+ plasma cells. These patterns have been labelled “pathotypes” and consist of (i) a “lympho-myeloid” pathotype, characterised by well-organised B-cell aggregates (called “ectopic lymphoid structures”), and often surrounded by plasma cells and abundant sublining macrophages, (ii) a diffuse-myeloid pathotype, characterised by a predominance of macrophages in the sublining but not B-cell follicles, and (iii) a pauci-immune/fibroid pathotype, characterised by a substantial lack of immune cell infiltration and abundant resident fibroblasts. Interestingly, the last pathotype had been previously seen in joint replacement tissue and was hypothesised to represent burnt-out disease, but it was instead demonstrated to also be present at the early stage of the disease and represented a distinct disease endotype.

When correlating histological pathotypes with RA autoantibodies in our cohort of early untreated RA patients, it was found that lympho-myeloid patients had significantly higher rates of RF (76.5%) and ACPA positivity (78.4%) and more active disease (highest swollen joint counts, CRP and erythrocyte sedimentation rate (ESR) and, consequently, highest DAS28). Conversely, the pauci-immune fibroid pathotype was the least associated with RF positivity (50%) or ACPA positivity (52.9%) and was characterised by significantly lower CRP and ESR in comparison with lympho-myeloid [155]. Further clinical relevance of the pathotype classification was found when correlating the pathotypes to longitudinal clinical data; patients with a lympho-myeloid pathotype were more likely to develop joint damage [155] and require escalation to biologics [156]. Of note, when assessing the relationship between histological pathotypes and response to treatment, patients with a fibroid/pauci-immune pathotype on synovial biopsy were less likely to achieve a response following treatment with certolizumab-pegol when compared to the lympho-myeloid or diffuse-myeloid pathotypes [157].

In a separate study, De Stefano et al. investigated differences in histological subtypes between seropositive and seronegative RA [15]. Analysing 131 synovial biopsies derived from seropositive RA, seronegative RA, oligoarticular psoriatic arthritis (PsA), and polyarticular PsA, they reported that lympho-myeloid synovitis and B-cell infiltration were increased in seropositive RA compared to seronegative RA. Interestingly, B-cell infiltration in seronegative RA was comparable to that observed in polyarticular psoriatic arthritis samples, suggesting that the histological stratification of patients may be helpful to refine the clinical classification.

These observations, combined with findings from our own group, are consistent with previous knowledge that RF/ACPA-positive patients, more often characterised by a lympho-myeloid pathotype, are characterised by a more severe disease and erosive burden. However, the significance of the lympho-myeloid pathotype in seronegative RA warrants further investigation regarding the underlying pathways. Moreover, it would be interesting to clarify the role of synovial ectopic lymphoid structures in those patients seronegative for RF and ACPAs but characterised by a lympho-myeloid pathotype.

#### 4.1.2. Focus on Ectopic Lymphoid Structures

As detailed in the previous section, one of the three pathotypes, i.e., the lympho-myeloid, is characterised by ectopic lymphoid structures (ELS). To offer better targeted treatments to RA patients, a deeper understanding of the pathogenesis of seropositive and seronegative RA is needed. Although the origin and development of ELS in RA synovial tissue is still not completely understood, they have the potential to explain, at least partially, antigen-driven responses in RA [158].

**Figure 1:**
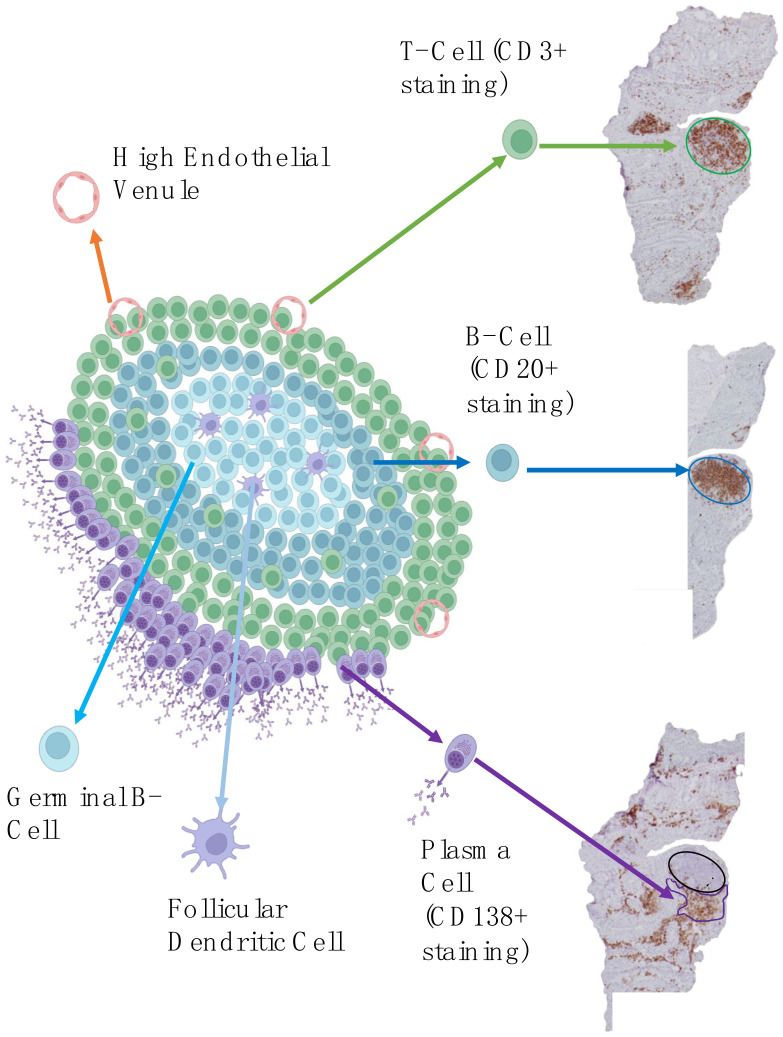
Ectopic lymphoid structure in rheumatoid arthritis. Schematic representation of an ectopic lymphoid structure (ELS) with corresponding histological images of an ELS within the synovial tissue formed by B cells (CD20+ staining) and T cells (CD3+ staining), and surrounded by plasma cells (CD138+ staining). Created with BioRender.com.

ELS are transient segregated clusters of T and B lymphocytes resembling lymphoid follicles found in secondary lymphoid organs but detectable in non-lymphoid organs, for example, the synovial tissue. They are characterised by high endothelial venules (HEVs) that allow L-selectin-expressing lymphocytes to pass from the blood into the tissue as well as a network of follicular dendritic cells (FDCs) [158]. Figure 1 shows the structure of ectopic lymphoid structures and how they appear on histological immunostaining.

ELS can resemble the germinal centres seen in secondary lymphoid organs such as the lymph nodes and spleen. Normally, in these germinal centres, B cells in the dark region of the follicle undergo somatic hypermutation, which allows the production of a diverse set of sequences coding for the variable region on immunoglobulins. B cells express the antibody on the cell surface and subsequently move to the light zone where they are presented with an antigen by follicular dendritic cells and interact with T-helper cells (Th cells). B cells that bind to the antigen strongly receive survivor signals and are selected for affinity mutation and subsequently differentiate into B memory cells or plasma cells. Class switching also occurs when B cells are activated, leading to the production of activation-induced cytidine deaminase, which converts cytosine to uracil at certain positions in the genome. This in turn changes the constant region of the antibody, leading to different types of antibodies being produced.

These structures can be found in chronically inflamed tissues in association with autoimmunity [159]. They have been detected in the synovium in RA [160], in salivary glands of patients with Sjogren’s [161], and in the kidneys of patients with systemic lupus erythematosus [162]. They have also been found in the synovium of osteoarthritis [163]. Several actors are involved in the development of ELS including the Il-23-IL 17 pathway [164,165], lymphoid tissue inducer cells [166], and chemokines such as CXCL13/CXCR5 [167], CCL19, and CCL21/CCR7 [168]. Whether these structures are a consequence of chronic hyperinflation or whether they are directly involved in the pathogenesis of autoimmune disease is still under debate.

In a cohort of early untreated RA, 40% of patients were found to have ELS, but the prevalence varies depending on the stage of disease, what treatment has been given, and technical factors such as staining and how the sample was collected [158,169,170,171,172]. Synovial ELS resembling germinal centres may be functional and contribute to the local production of autoantibodies such as ACPAs. This idea is supported by the fact that mice with severe combined immunodeficiency that are transplanted with human synovium can be found to produce ACPAs [172]. However, the authors of this study note that these findings do not rule out the possibility that B cells which are already mutated are recruited to the synovium.

The presence of ELS in synovial tissue has been associated with a reduced response to treatment and longer disease duration [173]. However, when patients had a good response to anti-TNF therapy, the presence of ELS reversed [173].

To our knowledge, the presence of ELS in seronegative RA has not been extensively studied. Further work is needed to establish whether the presence of ELS can delineate seronegative RA into distinct endotypes, and whether the novel antibodies previously reviewed are associated with the presence of ELS.

#### 4.1.3. Transcriptomic Phenotypes

Building on the concept of histological pathotypes, our group demonstrated that individual histological patterns were characterised by specific transcriptional endotypes. In an initial analysis of 87 early treatment-naïve RA, as expected, B cells and myeloid genes were highly expressed in the lympho-myeloid pathotype, whereas the myeloid pathotype had low B-cell expression and high myeloid gene expression [155].

Subsequent work by Lewis et al. based on RNA sequencing data [174] showed that ACPA titres correlated greatest with plasma cell gene modules, corroborating the notion of differentiation of plasma cells in the synovium being associated with autoantibody production. Plasma cell gene expression also correlated with power Doppler signal and synovial thickening measured on ultrasound. However, when analysing associations with radiographic evidence of damage, several cell gene modules were found to correlate, including CD4+ memory T cells, T cells, B cells, and plasma cells, overall suggesting that early damage in RA is potentially due to multiple immune cells infiltrating the synovium. When comparing ACPA-positive and ACPA-negative patients, greater expression of plasma cell genes such as LAMP5, EAF2, ODC1, and XBP1 was found in the ACPA-positive population [174].

Further advances in understanding the cellular and molecular composition of the rheumatoid synovium were derived from transcriptomic data of another large biopsy-based study, the “Rituximab versus tocilizumab in anti-TNF inadequate responder patients with rheumatoid arthritis” (R4-RA trial) [175]; this was the first randomised controlled biopsy-driven trial worldwide. Patients with RA who had not responded to at least one anti-TNF agent underwent a baseline US-guided synovial biopsy and were randomised to either rituximab or tocilizumab [175]. Interestingly, patients characterised by a low molecular B-cell signature had a significantly improved CDAI50% following treatment with tocilizumab compared to rituximab, overall suggesting that in-depth analysis of the synovial tissue may be used to inform treatment choices. Further analysis of this trial identified over 6000 genes significantly different between responders to rituximab and non-responders, and found a “fibroblast” signature in patients refractory to all medications [176].

More recently, the development of cell-type abundance phonotypes (CTAPs) offered a unique perspective on understanding the pathology underlying the spectrum of RA. Zhang et al. combined single-cell RNA sequencing with histology of synovial tissue from 79 RA donors to build a “cell atlas” of inflamed rheumatoid arthritis synovial tissue, comprising more than 314,000 cells [177]. From this, they identified six major cell-type abundance phenotypes (CTAPs) which reflected different levels of enrichment of specific cells: (i) endothelial, myeloid, and fibroblast cells (CTAP-EFM), (ii) fibroblast cells (CTAP-F), (iii) fibroblasts and T cells (CTAP-TF), (iv) comprising T and B cells (CTAP-TB), (v) myeloid and T cells (CTAP-TM), and (vi) myeloid cells (CTAP-M).

These CTAPs correlated with several metrics of RA. Histologically, CTAP scores were associated with the Krenn synovitis score, a well-known system to grade the presence of inflammation in the synovium (*p* = 0.0004) [178]. Transcriptionally, distinct CTAPs were associated with specific cytokines. For example, CTAP-TF was linked with interferon gamma and TNF; CTAP-TB with CXCL13L, which is involved in organising lymphoid tissues; and CTAP-M with angiogenic factors such as vascular endothelial growth factor A (VEGFA). Clinically, CTAPs were found to change depending on treatment exposure; notably, CTAP-F was associated with poor response to clinical treatment, in keeping with our observation in the R4RA cohort where a fibroblast signature identified patients refractory to multiple biologic agents.

Concerning associations between CTAPs and ACPA status, interestingly, CTAP-M had significantly lower titres of ACPAs, even when the analysis was confined to ACPA-positive patients only [155]. While further work is needed to understand the relationship between CTAPs and autoantibody production, advances in transcriptomics offer the opportunity to reclassify disease more accurately than by seropositive status alone. In Table 2, we summarise the differences between seropositive and seronegative RA documented in the current literature, including synovial histological and molecular differences.

### 4.2. Synovial Tissue in Other Seronegative Diseases

The lack of confirmatory antibodies for seronegative RA presents a diagnostic challenge, and there is a possibility that undifferentiated arthritis may be misdiagnosed as seronegative RA, when it may develop into one of the diseases included in the spectrum of peripheral spondyloarthritis (SpA), such as psoriatic arthritis (PsA) or Inflammatory Bowel Disease (IBD)-associated arthritis. In a large Finnish study with over 9000 seronegative RA patients, 245 patients were subsequently reclassified as having PsA and 33 as having IBD-associated arthritis [179].

Further understanding of the immunopathologic pathways underpinning inflammation may be used to enhance the identification of seronegative RA versus other seronegative diseases. There are three major types of cell-mediated immunity [180]: Type 1, involving the production of interferon (IFN) gamma and primarily directed at activating pathways to target intracellular organisms such as viruses and some microbes; Type 2, characterised by the production of IL-4, IL-5, and IL-13, which trigger mast cell activation and IgE production to target invading helminths and venom; and Type 3, recruiting innate lymphoid cells, cytotoxic T 17 and Th17 cells able to activate the IL-17/ IL 22 axis, which, in turn, stimulates innate immune cells (e.g., macrophages and neutrophils) to protect against extracellular organisms. Type 1 and Type 3 cell-mediated immunity have been the most implicated in joint inflammation [181]. However, distinct inflammatory pathways may represent the main driver in certain types of arthritis compared to others. For example, by investigating T-cell cytokine pathways, Hughes et al. found that cytotoxic Th17 cells were significantly higher in the synovial fluid of patients with undifferentiated early inflammatory arthritis who subsequently developed PsA or SpA in comparison with patients who developed seronegative RA. Conversely, CD8 T cells expressing IFN were significantly higher in seropositive RA compared with seronegative RA as well as PsA or SpA [182].

A recent study investigated the association of poor prognostic factors with the presence of ELS in synovial tissue from patients with RA or PsA [183]. It has been suggested that poor prognostic factors, such as the need for escalation to biological therapy or the presence of bone erosions, were significantly higher in the presence of ELS and CD15-positive cell infiltrates. Notably, RF or ACPAs were considered poor prognostic factors for RA; as discussed above, it is possible that a subset of so-called “seronegative” patients may be characterised by a similar poor prognosis, and, thus, further investigation is needed to understand if this subgroup is also characterised by a higher presence of ELS.

Th2 diseases associated with the activation of Type 2 immunity and mediated by IL-4/IL1-13, such as allergic asthma and atopic dermatitis, have been treated with biologic drugs blocking IL-4 [184,185]; these agents target and switch off Th2-induced inflammation. Interestingly, a number of patients treated with these drugs have developed psoriasis [186] as well as enthesitis and, in some cases, even seronegative arthritis [187]. These conditions are typically mediated by Th17-type inflammation; the authors suggested a novel potential link between IL-4/IL-13 and the Th17 axis, whereby the former exerts a protective role against the development of inflammation.

Bridgewood et al. had previously shown that myeloid cells within the enthesis produced IL-23 in response to lipopolysaccharide, but this was significantly reduced when co-stimulated with IL-13 or IL-4 [187,188]. This suggests that IL-4 and IL-13 may be involved in regulating the IL-23-IL-17 pathway. Furthermore, in the synovial tissue of RA and OA patients, IL-4 has been shown to reduce IL-1 levels and TNF-alpha levels [189].

**Figure 2:**
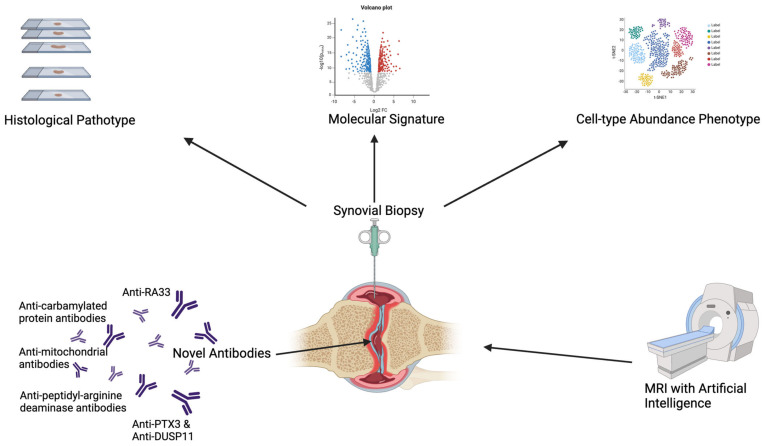
Methods to refine the classification of seronegative arthritis. Diagram conveying different methods that have the potential to delineate seronegative rheumatoid arthritis into distinct endotypes. Created in BioRender.com.

Overall, although further work is still needed, in-depth analysis of the synovial tissue may represent a promising tool to reveal unique or distinct endotypes that will enable better diagnostic certainty and facilitate targeted treatment, as summarised in Figure 2.

### 4.3. Other Models to Study Seropositive and Seronegative RA

To better understand the pathogenesis of RA, animal models have been widely used, with the majority of these being murine [190]. Examples of available tools to recapitulate some of the clinical features seen in RA in animal models include injecting subcutaneous Freund’s adjuvant to produce adjuvant-induced arthritis (AIA) [191], injecting type II collagen intradermally to produce collagen-induced arthritis (CIA) [190], or injecting type II collagen monoclonal antibodies to produce collagen antibody-induced arthritis (CAIA) [192,193]. CIA and CAIA differ in that T and B cells are required to induce CIA [194], whereas CAIA can be induced in the absence of T or B cells [192]. In view of the role of the adaptive immune system in seropositive RA and the potential link with synovial lymphoid structures, the CIA model could be considered a better model for seropositive RA [195]. Of interest, rhesus monkeys have been observed to develop synovitis similar to RA when injected with cyclic citrullinated vimentin [196], thus providing a good model for ACPA-positive RA.

These models are generally helpful for studying the effects of new drugs and immune responses [197], but, given the differences between the human and non-human immune systems, the validity and reliability of results will always be under scrutiny when translating research to human patients. A potential solution for assessing human tissue behaviour in an animal model is to use mice with severe combined immunodeficiency (SCID) which can be transplanted with synoviocytes or synovial tissue from patients with RA [195].

An alternative approach may be to use novel 3D cell culturing technology to develop synovial organoids. This is an emerging field and different techniques have been proposed, ranging from “bioprinting”, where cell layers are printed to construct three-dimensional structures that imitate human tissue [198], to developing three-dimensional “synovium-on-a-chip”, which can non-invasively monitor cell parameters [199].

In oncology, patient-derived organoids have been recently used to assess responses to therapy [200]. Reviewing recent advances in precision medicine in RA, Bhamidipati et al. [201] argue for developing synovial patient-derived organoids derived from synovial biopsies. This would enable targeted treatment that has been tested in vitro, and it would also allow us to understand the various pathogenic mechanisms in seropositive and seronegative RA.

## 5. Conclusions

RA patients are currently divided into “seropositive” and “seronegative” based on the presence of RF or ACPAs.

However, it has also been recognised that, due to the lack of confirmatory antibodies, seronegative RA patients represent a diagnostic challenge. Such diagnostic uncertainty, fuelled by the lack of solid biomarkers, may lead to delayed diagnosis and treatment escalation and has the potential to translate into a worse prognosis. To improve patient outcomes in seronegative RA, a better classification of the disease is needed to reduce diagnostic uncertainty and enable targeted treatment.

An obstacle in the path to treating RA optimally is the heterogeneity of the disease and the many possible immunological pathways that can be involved. Histological and molecular signatures from synovial biopsies offer a deeper understanding of the disease pathways, and most importantly, the ability to target treatment at the site of the inflammation. Clinical trials incorporating the use of synovial biopsies are ongoing and showing promising results, but further work is needed to understand if this “precision medicine” approach could be applied in routine practice.

Nevertheless, refining seronegative RA clinical classification by integrating molecular pathology and CTAPs into clinical algorithms will better define the characteristics of these patients and enable targeting of biologic therapies to specific pathways expressed differently in individuals.

## Figures and Tables

**Table 2:** Summary of differences between seropositive and seronegative RA from findings in the recent literature.

	Seropositive	Seronegative
Age at diagnosis [20]	43 ± 14 years	54 ± 11 years
Genetic and environmental factor differences [25,26,27,28,29,30]	- Associated with HLA-DRB1*04 and HLA-DRB1*10 haplotypes - Associated with variants of genes for the JAK/STAT pathway	- Associated with HLA-B08/DRB103 haplotype - SNPs in the CLYBL gene and ANKRD55 gene
Immunopathogenesis [21,36,37,42,51]	- Smoking and certain bacteria associated with periodontitis may be involved in citrullination, leading to target antibodies binding to citrullinated epitopes.	- Great numbers of M1-like pro-inflammatory macrophages - Macrophages and dendritic cells have significantly upregulated gene expression of CCL13, CCL18, and MMP3 - Increased levels of IL-6 instigating signalling of STAT3 in CD4+ T cells in early RA
Clinical features [61,62,63,64,68,84]	- Scleritis more likely to be present - Rheumatoid nodules more associated with seropositive RA - Significant association with radiographic progression on X-ray - Anti-CCP titres more associated with interstitial lung disease	- Methotrexate less effective - More associated with tenosynovitis
Synovial histological and molecular differences [15,155,174,177]	- Lympho-myeloid more associated with RF (76.5%) and ACPA (78.4%) positivity - ACPA titres correlate with plasma cell gene modules - Increased expression of plasma cell genes LAMP5, EAF2, ODC1, and XBP1	- B-cell infiltration in seronegative RA comparable to that observed in polyarticular psoriatic arthritis samples - Myeloid cell-type abundance phenotypes (CTAPs) have significantly lower titres of ACPAs

## Data Availability

No new data were created or presented in this manuscript.
